# Mallet-ing

**Published:** 1860-09

**Authors:** 


### MALLET-ING.

Of all the hobbys e'er bestrode
By doctor, priest or poet,
The very worst that e'er was rode,
And one that neither spur nor goad,
Or both combined, could force a pace
So fleet, that in an even race
A snail would not outgo it—
Is that old fogyish lumbering "notion,"
"Propelled by wife or helper's motion,"
Apostrophized "My Mallet!"
An implement of much avail,
When modeled on an ample scale,
For driving posts, or "mauling rails,"
To calkers, carpenters and tinners,
As necessary as their dinners;
And, therefore, were its praises sung,
The grateful Peans from among
The strong armed laborers should ascend
To hail the mallet; as a friend
Tried—trusty—indispensable!
But that ever a dentist should smuggle it in
To assist him in *filling*, with gold or with tin,
On sensitive dentine and that very thin—
('Twixt the plugger and nerve) is so serious a sin—
Against reason, and judgment, and good common sense,
As to merit the verdict of "malice prepense."
But dentists there are
Who boldly declare
That the maul-it in process—or whate'er they call it
The difference is trifling 'twixt mallet and maul it—
Is good for the patient, quite mild on "mouth tissue,"
Is safe for the tooth,—and if in the issue,
Brain, tissue, and tooth survive the confusion;
Why—the method is preferable far to the rushing
Of hand force in packing a carious stump,
By the difference in pain—between a push and a thump—
Then "*try* it profession, before you abuse it;"
Try it all ye who have wives to help use it;
Bachelors try it! no fear you'll miscarry,
Should a helper be needed, you surely can marry!
Try it on soft foil, adhesive, or crystal;
If the mallet should fail, shoot it in with a pistol;
For the patient whose nerves are proof to the one
Is not likely to flinch should you fill with a gun!—E. J. W.

